# Correlation Between Socioeconomic Status and Brace Compliance in Idiopathic Clubfoot Deformities

**DOI:** 10.7759/cureus.80254

**Published:** 2025-03-08

**Authors:** Ennio Rizzo Esposito, Rachel Phillips, Emily V Leary, Sumit K Gupta

**Affiliations:** 1 Orthopedic Surgery, University of Missouri, Columbia, USA

**Keywords:** bracing, clubfoot, compliance, deformities, socioeconomic status

## Abstract

Background: Congenital talipes equinovarus (CTEV), or clubfoot, is one of the most common foot deformities seen at birth. The Ponseti technique is the most common method to treat clubfoot and consists of gentle manipulation with serial casting, a percutaneous Achilles tendon tenotomy, and bracing for the first few years of life. The purpose of this study was to determine whether socioeconomic factors influence compliance with clubfoot bracing for families with infants who have idiopathic clubfoot treated by the Ponseti method.

Methodology: All patients with clubfoot deformity who began primary treatment at our pediatric orthopedic clinic between February 2018 and May 2021 were included in a retrospective chart review. Compliance was defined as strict adherence to the initial casting and tenotomy appointments, in addition to the caregiver's reported compliance with brace wear, and no mention of non-compliance in the patient’s medical record. Recurrence was defined as relapse of the deformity after at least one year of follow-up and/or the need for additional casting or tenotomy.

Results: Forty-three patients were included in the final analysis of compliance with bracing. No significant correlations were seen between the rate of compliance with bracing and any of the socioeconomic factors assessed in this study. The odds of noncompliance were 7.0 times higher in patients who had one or more missed clinic appointments, compared to those who attended all appointments (*P *= 0.01). Forty-one patients were analyzed at a minimum one-year follow-up for recurrence of deformity. The odds ratio for recurrence of deformity in patients who were noncompliant with bracing was 74.8 compared to those who were compliant (*P *=< 0.001).

Conclusions: Socioeconomic factors, including household income, education level, zip code, employment status of caregivers, and insurance status, were not associated with bracing compliance or recurrence of the clubfoot deformity. This was a level 2 observational study.

## Introduction

Congenital talipes equinovarus (CTEV) deformity, also known as clubfoot deformity, is one of the most common congenital foot deformities, with a rate of 1 in 1,000 live births per year in the United States [1]. Clubfoot is a complex, three-dimensional deformity characterized by pes cavus, varus, adductus, and equinus. The severity of the deformity varies from patient to patient [2,3]. Patients with untreated clubfoot will bear weight on the lateral or dorsal surface of the foot, which can lead to hyperkeratosis, potential skin compromise, altered gait pattern, pain, and difficulty with shoe wear [4]. Fifty percent of cases occur bilaterally, and 70% occur in males [5].

Since 1963, Ponseti and others have shown that a combination of gentle manipulation, placement of well-shaped corrective casts, and percutaneous tenotomy of the Achilles tendon can successfully treat clubfoot deformity while avoiding major surgical intervention [6,7]. Numerous studies have demonstrated short- and long-term success, with recurrence rates ranging from 5% to 7% two years after casting [3,8].

Recurrence rates are closely related to compliance with casting and bracing throughout the treatment period. Using foot abduction braces (FABs) after initial correction is key to avoiding recurrence. The braces should be worn for 23 hours per day for the first three months, followed by night- and naptime wear for three to four years. Adherence to this protocol requires significant dedication from the family. Several studies have shown a correlation between noncompliance and recurrence of deformity [1,5-7,9-11]. However, the underlying reasons leading to noncompliance remain unclear in the literature.

Few studies have investigated the effect of socioeconomic factors on brace compliance and outcomes of clubfoot. Avilucea et al. [12] analyzed 138 clubfeet treated with the Ponseti method and found that risk factors for noncompliance with bracing included living in a rural area, Native American ethnicity, unmarried parents, insurance status, and family income of less than $20,000. Dobbs et al. [8] reported that parental educational level was a significant risk factor for recurrence (odds ratio = 10.7, *P* < 0.03). However, they did not find any correlation with other factors, including gender, race, parental marital status, type of medical insurance, or parental income. Ramirez et al. [13] in their study of 73 clubfeet reported a recurrence rate of 33% but found no association with patient gender, age at presentation, cast treatment duration, laterality, severity, or family educational or income level.

The effect of socioeconomic factors on outcomes of clubfoot treatment remains unclear. This study aimed to determine if socioeconomic factors, including household income, caregiver education level, employment status, insurance status, zip code, and gender, affect the rate of clubfoot bracing compliance or recurrence of deformity in patients with idiopathic clubfoot treated at our institution.

## Materials and methods

After Institutional Review Board (IRB) approval, we identified all patients with clubfoot who initiated treatment at our pediatric orthopedic clinic between February 2018 and May 2021. Demographic information (age, gender, type of insurance, and zip code) was extracted from patients' medical records. Socioeconomic data (household income, parental education, employment, and marital status) were extracted from self-reported patient intake forms completed at the initial visit. Any missing data for household income were imputed using publicly available mean income data based on zip codes.

Patients were excluded if they received their initial treatment elsewhere, did not have a diagnosis of congenital clubfoot deformity (i.e., congenital vertical talus [CVT], positional clubfoot), or had a syndromic or neuromuscular etiology. Patients with less than one year of clinical follow-up were included only in the analysis for brace compliance but excluded from the recurrence analysis. 

The treatment protocol of casting and bracing was similar to that described by Ponseti, and all procedures were completed by the senior author of this paper. All patients underwent weekly manipulation and casting, followed by percutaneous Achilles tendon tenotomy. After final cast removal, patients were braced for 23 hours per day for three months and then at nighttime only for four to five years. 

Compliance was defined as strict adherence to casting and initial Achilles tendon tenotomy appointments in our clinic during the initial treatment, and parent-reported compliance during the bracing period. The medical record was searched for any mention of missed appointments or documented noncompliance. Recurrence was defined as relapse of the deformity after at least one year of follow-up and/or the need for additional casting, tenotomy, or other surgical procedures such as anterior tibialis tendon transfer.

Statistical analysis

Chi-square and Fisher’s exact tests were employed to calculate odds ratios with 95% confidence intervals (CIs) for compliance with bracing and recurrence of deformity for all categorical variables. Mann-Whitney U and t-tests were utilized to assess differences in means or medians for continuous variables. The significance level was set at *P* < 0.05 for all analyses. All statistical analyses were conducted using R version 4.2.1.

## Results

**Figure 1:**
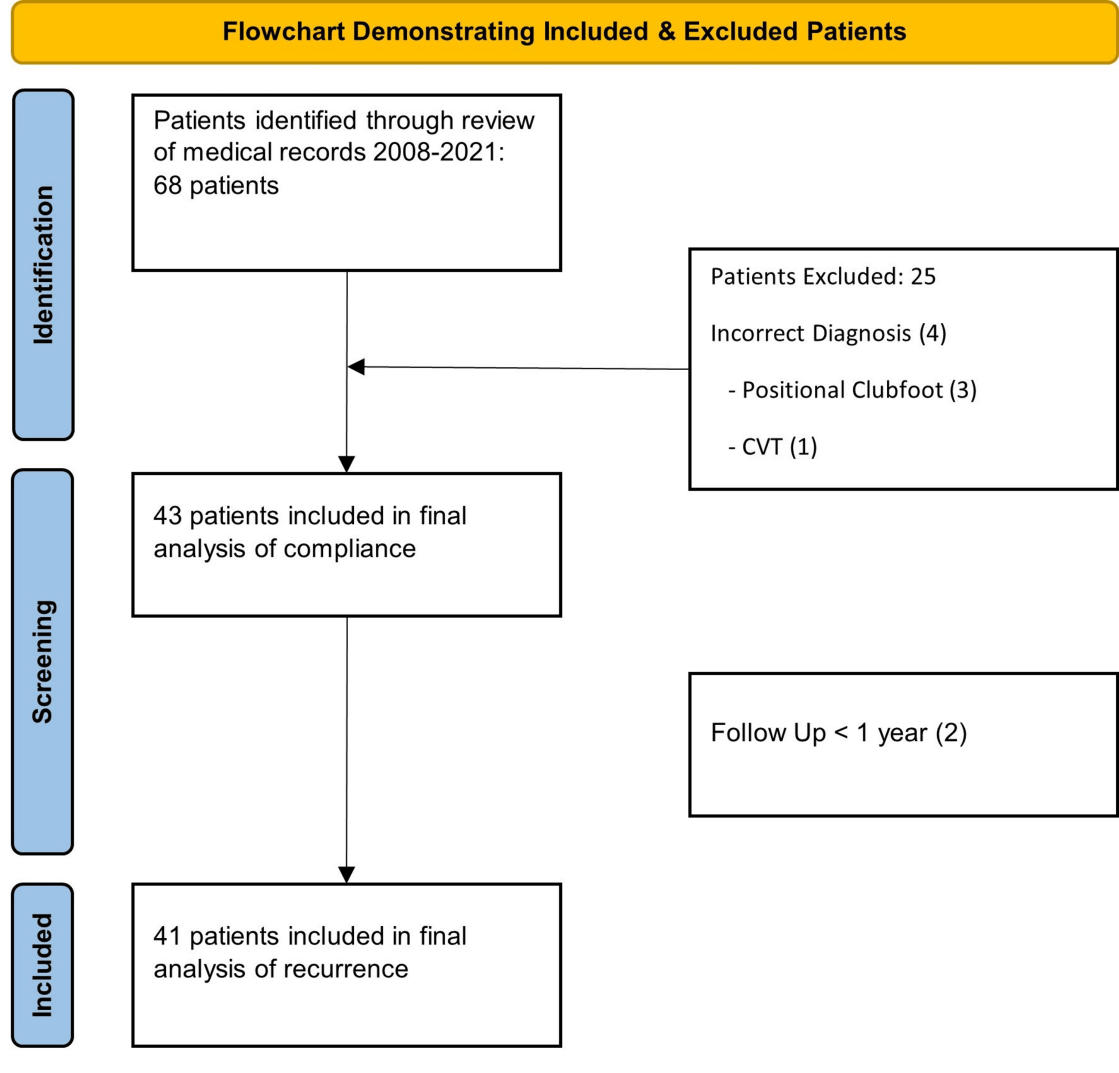
Flowchart demonstrating inclusion and exclusion reasons. Image credit: Ennio Rizzo Esposito and Sumit K. Gupta.

In total, 68 patients were treated in our clinic for clubfoot deformity between February 2018 and May 2021. After exclusions, 43 patients were included in the final analysis for bracing compliance (Figure 1).

Overall, 17 (40%) were girls and 26 (60%) were boys. Among them, 17 (40%) patients had bilateral clubfeet, 12 (28%) had left-sided, and 14 (32%) had right-sided clubfoot (Table 1).

**Table 1:** Demographic information.

Demographics	
Females	20
Males	23
Bilateral clubfeet	17
Left clubfoot	12
Right clubfoot	14

The median household income for self-reported income fell within the $10,001-$25,000 category; however, 23 families chose not to report their income on our patient intake forms. For those 23 families, income levels were imputed using nationally published data on mean income by zip code, resulting in a median income level of $25,001-$50,000 for the complete data set. In total, 25 families reported the highest education level of the caregivers, with 14 (56%) having a high school or lower education level. Of the total, 50% of patients had public insurance and 50% had private insurance. The caregivers' employment status was reported by 31 families, with 3 (10%) indicating unemployment. Additionally, 34 families reported their marital status, with 24 (71%) families being married or otherwise living together, and 10 (29%) being single-parent households. Out of the 43 patients, 41 had at least one year of follow-up and were included in the recurrence analysis (Figure 1).

Overall, 27 (62.8%) patients were compliant with bracing, while 16 (37.2%) patients were noncompliant. Tables 2-3 demonstrate the demographic and socioeconomic factors associated with compliance. The only relationship observed was between missed appointments and brace compliance. Compliant patients had an average attendance rate to clinic appointments of 96.5%, compared to 89.4% for noncompliant patients (*P *= 0.0046). The odds of noncompliance were seven times higher for those who missed appointments compared to those who did not (95% CI 1.44-47.75). No statistically significant difference was observed in any of the factors when considering compliance.

**Table 2:** Categorical variables and associations with brace compliance. OR, odds ratio; CI, confidence interval; GI, gastrointestinal; NA, not applicable

Independent variable	Category	Noncompliant	Compliant	ORs	95% CIs	*P*-value
Household income (Imputed)	$10,001-$25,000	5	6	-	-	0.698
	$25,001-$50,000	5	9	1.5	0.299-7.531	
	$50,001-$100,000	5	11	1.83	0.374-8.985	
		1	0	0.467	0.011-15.193	
	>$100,000	0	1	1.667	0.0457-60.772	
Household income	Missing	9	14	-	-	0.733
	$10,001-$25,000	4	6	0.964	0.212-4.397	
	$50,001-$100,000	2	6	1.929	0.317-11.740	
		1	0	0.321	0.0097-10.608	
	>$100,000	0	1	1.286	0.039-42.432	
Insurance type	Medicaid	10	9	3.111	-	0.184
	Private	5	14	-	0.797-12.140	
Marital status of the caregiver	Married	9	15	-	-	1
	Single	3	7	1.4	0.287-6.831	
Employment status of the caregiver	Not working	1	2	-	-	1
	Working	9	19	1.056	0.084-13.226	
Education level of the caregiver	Associate degree	0	1	-	-	0.389
	Bachelor's degree	1	2	1	0.016-63.930	
	High school/GED	5	6	0.6	0.017-21.878	
	Less than high school	3	2	0.333	0.007-15.469	
	Postgraduate training	0	5	5	0.057-436.54	
	Vocational/Trade school	1	1	0.5	0.006-40.027	
Comorbidity type	Cardiac	1	0	-	-	0.405
	Cardiac, GI	0	1	4	0.033-486.53	
	GI	0	2	8	0.081-793.18	
	NA	13	19	2.923	0.091-93.689	
	Renal	0	1	4	0.033-486.53	
	Respiratory	1	0	1	-	
Comorbidities? (Y/N)	No	13	19	-	-	1
	Yes	2	4	1.368	0.218-8.601	
Delivery method	C-section	6	11	-	-	0.980
	Vaginal	9	13	0.788	0.213-2.915	
Birth presentation	Breech	9	9	-	-	1
	Cephalad	5	13	0.963	0.253-3.67	
Gender	Female	5	12	-	-	0.594
	Male	11	15	0.568	0.155-2.088	
Race	African American	1	0	-	-	0.372
	White	15	27	3.6	0.114-113.74	
Additional casting after tenotomy? (Y/N)	No	9	23	-	-	0.068
	Yes	7	4	0.224	0.052-0.953	
Additional tenotomy? (Y/N)	No	10	23	-	-	0.137
	Yes	6	4	0.290	0.067-1.257	
Other procedure? (Y/N)	No	10	23	-	-	0.137
	Yes	6	4	0.290	0.067-1.257	
ATT? (Y/N)	No	11	25	-	-	0.0822
	Yes	5	2	0176	0.03-1.051	
Diagnosis	Bilateral	8	9	-	-	0.476
	Left clubfoot	3	9	2.667	0.529-13.433	
	Right clubfoot	5	9	1.6	0.375-6.820	
Household income (Categorical)		11	15	-	-	0.594
	>$50,000	5	12	1.76	0.480-6.467	
Diagnosis (Categorical)	Bilateral clubfeet	8	9	-	-	0.449
	Single clubfoot	8	18	2	0.564-7.087	
Education level of the caregiver	High school or less	8	8	-	-	0.124
	More than high school	2	9	4.5	0.73-27.740	

**Table 3:** Continuous variables and associations with brace compliance. IQR, interquartile range; SD, standard deviation

Independent variable	Noncompliant				Compliant				*P*-value
	Mean	SD	Median	IQR	Mean	SD	Median	IQR	
Median income ($)	46265.62	10060.93	44396	41081-49521	47306.27	9419.02	44730.5	39736.5-53127.5	0.99
Length of nursery stay	7.14	3.25	7.5	7-9.75	6.48	4.58	6	2-9	0.575
Birth weight (lbs.)	11.06	7.05	8	6.75-17.25	14.59	7.68	13.5	8.5-21	0.152
Length of follow-up (Days)	1113.69	786.28	948.5	507-1370.5	1272.37	958.86	1042	518.5-1583.5	0.737
Age at last follow-up (Days)	1166.44	767.72	1001.5	624.5-1409	1306.19	965.95	1100	537-1609.5	0.970
Number of additional casts after tenotomy	2.12	2.6	0	0-5	0.33	1.04	0	0-0	0.0182
Rate of attendance (%)	89.38	10.13	91.81	88.09-95.16	96.54	5.47	100	93.8-100	0.0046
Total ortho appt no-shows	2	2.28	1	1-2.25	0.48	0.85	0	0-1	0.0009
Total appts w/Gupta	17.94	8.47	17.5	12.75-21.25	15.22	5.49	14	11.5-17.5	0.2365
Time from initial presentation to tenotomy (Days)	9.19	5.97	9	5-14.5	7.52	5	6	2.5-11.5	0.4397
Number of casts required (before and day of tenotomy)	4.75	1.34	5	4-6	4.78	1.25	5	4-6	0.8654
Age at initial presentation (Days)	52.75	119.28	17.5	14.5-33.25	33.81	47.09	17	11-37	0.8406

Recurrence of deformity was observed in 17 (41.5%) patients. Tables 4-5 show the demographic and socioeconomic factors associated with recurrence. In total, 13 out of 17 patients (76.5%) with recurrence were not compliant with bracing, resulting in an odds ratio of 0.02 for recurrence in compliant patients compared to noncompliant ones (95% CI 0.0-0.15). No other statistically significant relationships were observed in any of the factors with recurrence.

**Table 4:** Categorical variables and associations with recurrence. OR, odds ratio; CI, confidence interval; GI, gastrointestinal; NA, not applicable

Independent variable	Category	No recurrence	Recurrence	ORs	95% CIs	*P*-value
Household income (imputed)	$10,001-$25,000	4	7	-	-	0.158
	$25,001-$50,000	8	5	0.357	0.068-1.88	
	$50,001-$100,000	11	4	0.208	0.039-1.114	
		0	1	1.143	0.031-42.26	
	>$100,000	1	0	0.286	0.008-10.57	
Household income	Missing	13	8	-	-	0.358
	$10,001-$25,000	4	6	2.438	0.522-11.39	
	$50,001-$100,000	6	2	0.542	0.087-3.37	
		0	1	3.25	0.097-108.4	
	>$100,000	1	0	0.813	0.024-27.10	
Insurance type	Medicaid	7	10	-	-	0.102
	Private	14	5	0.25	0.061-1.020	
Marital status of the caregiver	Married	15	8	-	-	1
	Single	6	3	0.938	0.184-4.79	
Employment status of the caregiver	Not working	1	2	-	-	0.251
	Working	19	8	0.211	0.017-2.67	
The education level of the caregiver	Associate degree	1	0	-	-	0.522
	Bachelor's degree	2	1	1	0.016-63.93	
	High school/GED	6	4	1.333	0.036-49.93	
	Less than high school	1	3	6	0.101-354.9	
	Postgraduate Training	5	1	0.4	0.007-22.21	
	Vocational/Trade school	1	0	1	0-0	
Comorbidity type	Cardiac	0	0	-	-	0.498
	Cardiac-GI	1	0	0.5	0.003-89.35	
	GI	2	0	0.25	0.002-36.99	
	NA	17	14	0.824	0.015-44.22	
	Renal	1	0	0.5	0.003-89.35	
	Respiratory	0	1	2	0.01-357.39	
Comorbidities? (Y/N)	No	17	14	-	-	0.376
	Yes	4	1	0.304	0.030-3.036	
Delivery method	C-section	11	6	-	-	0.792
	Vaginal	11	9	1.5	0.397-5.66	
Birth presentation	Breech	9	6	-	-	1
	Cephalad	11	9	1.228	0.316-4.770	
Gender	Female	9	9	-	-	0.508
	Male	15	8	0.533	0.151-1.88	
Race	African American	0	1	-	-	0.415
	White	24	16	0.333	0.011-10.53	
Additional casting after tenotomy? (y/n)	No	23	7	-	-	0.0004
	Yes	1	10	32.86	3.56-303.43	
Additional tenotomy? (y/n)	No	23	8	-	-	0.0005
	Yes	1	9	25.88	2.82-237.56	
Other procedure? (y/n)	No	23	8	-	-	0.0005
	Yes	1	9	25.88	2.82-237.56	
ATT? (Y/N)	No	23	11	-	-	0.0141
	Yes	1	6	12.55	1.34-117.33	
Diagnosis	Bilateral	8	10	-	-	0.285
	Left clubfoot	8	4	0.4	0.088-1.826	
	Right clubfoot	8	3	0.3	0.059-1.516	
Household income (categorical)	Less than $50,000	12	13	-	-	0.113
	More than $50,000	12	4	0.308	0.078-1.219	
Diagnosis (categorical)	Bilateral clubfeet	8	10	-	-	0.193
	Single clubfoot	16	7	0.35	0.097-1.27	
The education level of the caregiver	High school or less	7	7	-	-	0.208
	More than high school	9	2	0.222	0.035-1.42	

**Table 5:** Continuous variables and associations with recurrence. IQR, interquartile range; SD, standard deviation

Independent variable	No recurrence				Recurrence				*P*-value
	Mean	SD	Median	IQR	Mean	SD	Median	IQR	
Median income ($)	47887.26	9533.25	45363	40241-54417.5	45855.47	9998.37	44359	41089-46792	0.692
Length of nursery stay	6.39	4.2	6.5	2.25-9	7.27	3.97	8	4.5-9.5	0.543
Birth weight (lbs.)	15.2	8.22	13.5	9.5-22.25	11.5	7.36	8	7-18	0.164
Length of follow-up (Days)	1207.96	945.44	1016.5	521.75-1342.5	1329.88	839.69	1238	637-2037	0.487
Age at last follow-up (Days)	1234.92	938.29	1083	538-1360.25	1364.65	865.1	1255	659-2053	0.455
Number of additional casts after tenotomy	0.08	0.41	0	0-0	2.41	2.53	1	0-5	0.0001
Rate of attendance (%)	95.76	5.66	100	92.15-100	92.87	9.35	95	90.91-100	0.368
Total ortho appt no-shows	0.58	0.88	0	0-1	1.24	1.44	1	0-2	0.134
Total appts w/Gupta	14.92	5.87	13	11-18	18.24	7.7	17	15-21	0.0594
Time from initial presentation to tenotomy (Days)	7.96	5.54	6	2-12	9.41	5.68	9	5-14	0.415
Number of casts required (before and day of tenotomy)	4.83	1.17	5	4-6	4.82	1.42	5	4-6	0.620
Age at initial presentation (Days)	23.96	24.64	17.5	11-36	34.76	55.34	17	15-32	0.989

## Discussion

CTEV deformity is a prevalent congenital foot anomaly, affecting around 1 in 1,000 live births annually in the United States. It manifests as a complex foot deformity involving pes cavus, varus, adductus, and equinus, with severity varying among individuals. The Ponseti method has been a successful non-surgical treatment, emphasizing gentle manipulation, corrective casting, and Achilles tendon tenotomy. Compliance with casting and bracing is critical for preventing recurrence. Socioeconomic factors may influence compliance and recurrence rates, but findings are inconsistent in the literature.

The socioeconomic factors assessed in our study, including gender, race, household income, parental education levels, type of insurance, and marital status, showed no association with bracing compliance or recurrence of deformity in children with clubfoot managed using the Ponseti method.

Ramirez et al. [13], in their series of 73 clubfeet, did not find a correlation between bracing compliance rates and parental education level or type of insurance. Dobbs et al. [8], in a retrospective series of 51 children (86 feet), reported no statistically significant relationship between parental marital status, source of medical insurance, or parental income, and the risk of clubfoot deformity recurrence. However, they did find that a parental education level of less than high school was correlated with an increased risk of recurrence. Chong et al. [14] conducted a prospective, randomized trial with 30 consecutive patients treated for idiopathic clubfeet. In this series, mean income, education level, and age of caregiver tended to be lower in the recurrence group but were not statistically significant.

Akinyoola et al. [15] used a different approach to assess the socioeconomic status of the children in their clubfoot study. They utilized the Area Deprivation Index (ADI), a comprehensive measure of economic disadvantage calculated using 17 variables and based on the neighborhood of residence. They assessed 168 children and did not find any correlation between ADI and recurrence of deformity. 

Some studies have shown results that seem to contradict our findings. Avilucea et al. [12], in a prospective series of 138 clubfeet treated with the Ponseti method, reported that Native American ethnicity, unmarried parents, having public or no insurance, parental education at the high school level or less, and a family income of less than $20,000 were significant risk factors for recurrence in patients living in rural areas of America. However, their patient population was very different from the other studies mentioned above. They examined patients from the state of New Mexico, which has a population of over 50% non-White individuals, with most patients living in rural areas, leading to long commutes to healthcare facilities. Their findings may not apply to our patient population, which differs geographically, ethnically, and culturally.

Our study found that patients with missed clinic appointments had a significantly higher risk of noncompliance with bracing (OR 7.0, *P *= 0.01). This makes intuitive sense, as the factors responsible for noncompliance are likely the same ones that influence adherence to clinic visits. Missed appointments should therefore be seen as a red flag as these patients are at the highest risk for noncompliance.

Many studies have shown that noncompliance with the casting and foot abduction brace protocol in the Ponseti method is the main cause of relapse and has a direct effect on treatment success. This is consistent with the findings of our study, in which 76.5% of patients with recurrence were not compliant with bracing, resulting in an odds ratio of 0.02 for recurrence for compliant patients compared to noncompliant ones (95% CI 0.0-0.15). In a study conducted by Haft et al. [16], the noncompliance rate was 49%, and patients who did not adhere to the casting protocol were five times more likely to develop recurrence than compliant patients. In a series of 115 patients treated for clubfoot, Azarpira et al. [17] reported a noncompliance rate of 30%. Dobbs et al. [8] described a noncompliance rate of 41%, and they found that children who discontinued casting were 183 times more likely to have clubfoot recurrence. Masrouha and Morcuende [18] reported that noncompliance was associated with a 17-fold increased likelihood of relapse compared to compliance.

The socioeconomic factors assessed in this study showed no association with bracing compliance or recurrence of deformity in children with clubfoot managed using the Ponseti method. These findings support those of many other authors such as Ramírez et al. [13], Dobbs et al. [8], Chong et al. [14], and Akinyoola et al. [15]. However, some of the literature conflicts with our results, as described by Avilucea et al. [12]

Noncompliance with casting and bracing was the main cause of deformity relapse in our study. Our findings align with those previously described in the literature by many other authors.

There were many challenges in this study. Socioeconomic status was determined by optional disclosure from families on patient intake forms, leading to missing data that affected the power of the analysis. In addition, information bias may exist, as patients may be unwilling to indicate if they have lower education or income. The overall numbers in this study were low and may not have been sufficient to identify relationships between the variables and outcomes.

The exact definitions for noncompliance and recurrence are unclear in the literature, and different studies have used variable criteria to define these. Noncompliance was considered as complete discontinuation of bracing in some studies [8,12], less than 80% of nights in another study [13], and a caregiver statement or physician opinion in others [14,15]. Similarly, definitions of recurrence included the need for revision surgery, casting, or tenotomy in some studies [12,13,15], but relapse of hindfoot varus or ankle dorsiflexion in others [8,14]. Meaningful comparisons of these studies become difficult given the variability in definitions and outcomes, making it challenging to understand the true correlation between socioeconomic factors, compliance, and recurrence [19].

Our study was also limited in its ability to incorporate how cultural beliefs, ethnic background, and societal norms play a role in shaping attitudes toward healthcare and treatment adherence. Understanding these cultural factors could provide valuable context for interpreting compliance behaviors among diverse patient populations. Similarly, psychological factors such as parental stress, coping mechanisms, and perceptions of treatment efficacy were not included. Future studies incorporating behavioral interventions targeting these factors could enhance patient engagement and improve treatment outcomes.

Access barriers, including geographical constraints, transportation issues, and healthcare disparities, as described above, impact both compliance with appointments and long-term follow-up care. Future studies should address these access challenges as essential for ensuring equitable access to quality healthcare services for the management of this pathology.

Longitudinal studies tracking patients over extended periods are necessary to assess the sustained impact of socioeconomic factors on treatment outcomes. Long-term follow-up would allow for the identification of recurrence patterns and the evaluation of interventions aimed at mitigating risk factors for deformity recurrence in this patient population. Similarly, identifying avenues for future research, such as qualitative studies exploring the lived experiences of families managing clubfoot, investigating innovative interventions to enhance compliance, and assessing the impact of emerging technologies on remote monitoring and support, can advance our understanding of the complex interplay between socioeconomic factors and treatment outcomes.

## Conclusions

Socioeconomic factors, including income, education, type of insurance, employment, marital status, race, and gender, did not affect the brace compliance rates in our study. Noncompliance with clubfoot bracing is the most important reason for the recurrence of clubfoot deformity after treatment by the Ponseti technique. Any missed appointments should raise suspicion of non-compliance with treatment. A family's ability and willingness to adhere to the bracing recommendations are influenced by many factors, and more focused research is needed to identify these factors.
